# Supplementary material for: Novel Driver Strength Index highlights important cancer genes in TCGA PanCanAtlas patients
Source: PeerJ. 2022 Aug 11;10:e13860. doi: 10.7717/peerj.13860 (PMC9375969; doi:10.7717/peerj.13860)
Supplement: Supplemental Information 2 [file peerj-10-13860-s002.zip › COHORTS/genes plots/detailed/2021_8_16_14_9_distribution_events_detailed_16.pdf]

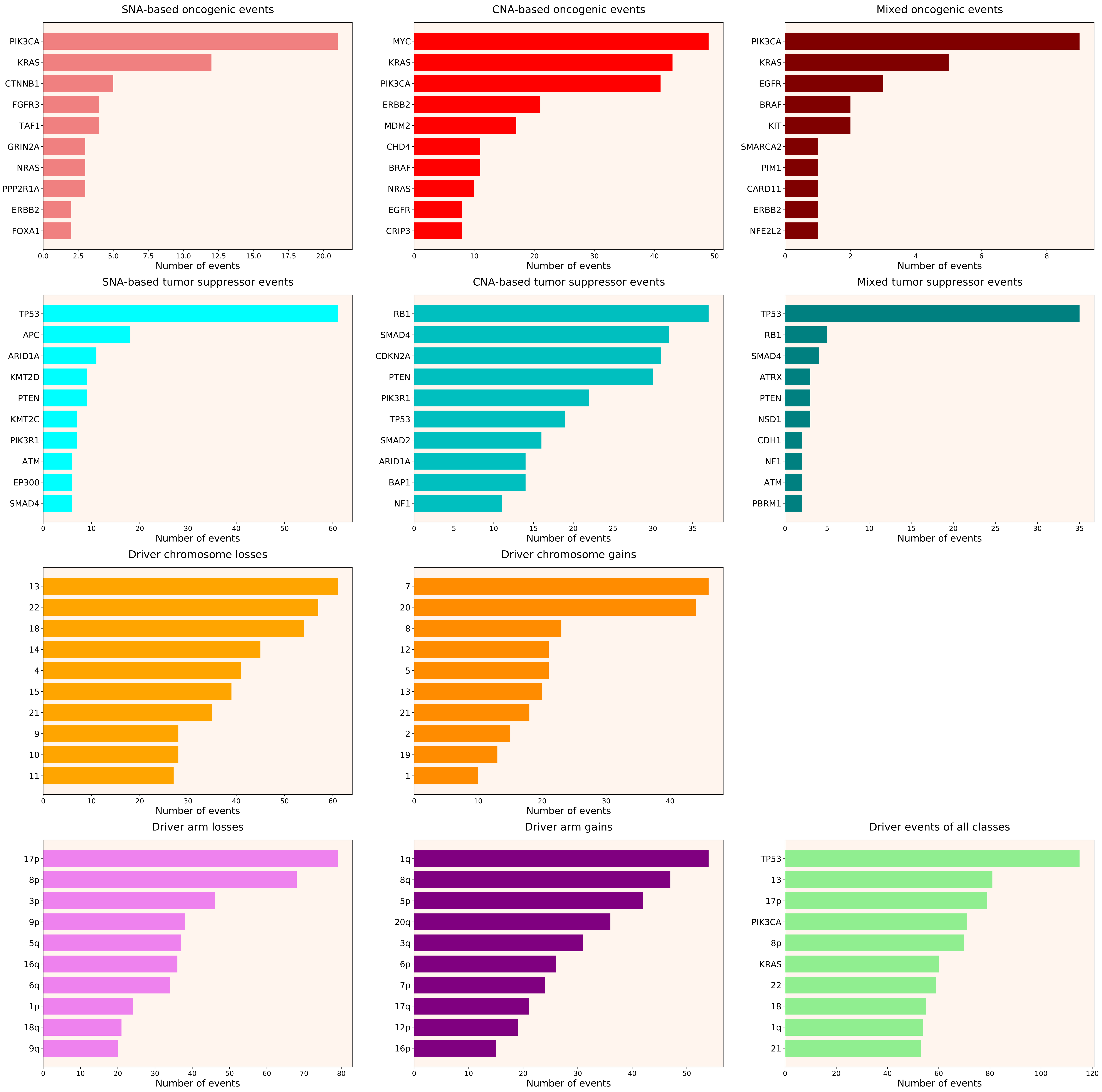

SNA-based oncogenic events
PIK3CA
KRAS
CTNNB1
FGFR3
TAF1
GRIN2A
NRAS
PPP2R1A
ERBB2
FOXA1
0.0 2.5 5.0 7.5 10.0 12.5 15.0 17.5 20.0
Number of events
CNA-based oncogenic events
MYC
KRAS
PIK3CA
ERBB2
MDM2
CHD4
BRAF
NRAS
EGFR
CRIP3
0 10 20 30 40 50
Number of events
Mixed oncogenic events
PIK3CA
KRAS
EGFR
BRAF
KIT
SMARCA2
PIM1
CARD11
ERBB2
NFE2L2
0 2 4 6 8
Number of events
SNA-based tumor suppressor events
TP53
APC
ARID1A
KMT2D
PTEN
KMT2C
PIK3R1
ATM
EP300
SMAD4
0 10 20 30 40 50 60
Number of events
CNA-based tumor suppressor events
RB1
SMAD4
CDKN2A
PTEN
PIK3R1
TP53
SMAD2
ARID1A
BAP1
NF1
0 5 10 15 20 25 30 35
Number of events
Mixed tumor suppressor events
TP53
RB1
SMAD4
ATRX
PTEN
NSD1
CDH1
NF1
ATM
PBRM1
0 5 10 15 20 25 30 35
Number of events
Driver chromosome losses
13
22
18
14
4
15
21
9
10
11
0 10 20 30 40 50 60
Number of events
Driver chromosome gains
7
20
8
12
5
13
21
2
19
1
0 10 20 30 40
Number of events
Driver arm losses
17p
8p
3p
9p
5q
16q
6q
1p
18q
9q
0 10 20 30 40 50 60 70 80
Number of events
Driver arm gains
1q
8q
5p
20q
3q
6p
7p
17q
12p
16p
0 10 20 30 40 50
Number of events
Driver events of all classes
TP53
13
17p
PIK3CA
8p
KRAS
22
18
1q
21
0 20 40 60 80 100 120
Number of events
